# 781. COVID-19 Outcomes in the Immunocompromised Population of the COVID-19 Community Research Partnership

**DOI:** 10.1093/ofid/ofac492.042

**Published:** 2022-12-15

**Authors:** John C Williamson, Gregory B Strylewicz, Michael E DeWitt, Diane Uschner, Ashvi Soni, Morgana Mongraw-Chaffin, Keerti L Dantuluri, Amy Hinkelman, Michael A Gibbs, William H Lagarde, William Weintraub, Matthew Bott, Brian Ostasiewski, Kristen Miller, Lewis McCurdy, John W Sanders, David M Herrington

**Affiliations:** Atrium Health Wake Forest Baptist, Winston-Salem, NC; The George Washington University, Manzanita, Oregon; Atrium Wake Forest Baptist Health/ Wake Forest University School of Medicine, Winston-Salem, North Carolina; The George Washington University, Manzanita, Oregon; The Biostatistics Center, The George Washington University, Washington, District of Columbia; Wake Forest School of Medicine, Winston-Salem, North Carolina; Levine Children's Hospital at Atrium Health, Charlotte, North Carolina; Campbell University School of Osteopathic Medicine, Lillington, North Carolina; Atrium Health - Carolinas Medical Center, Charlotte, North Carolina; WakeMed Health and Hospitals, Raleigh, North Carolina; MedStar Health Research Institute and Georgetown University, Washington, District of Columbia; George Washington University, Bethesda, Maryland; Wake Forest School of Medicine, Winston-Salem, North Carolina; MedStar Health National Center for Human Factors in Healthcare, Washington, District of Columbia; Atrium Health, Charlotte, North Carolina; Wake Forest University School of Medicine, Winston-Salem, North Carolina; Wake Forest university School of Medicne, Winston Salem, North Carolina

## Abstract

**Background:**

The COVID-19 Community Research Partnership (CCRP) is a large multicenter healthcare system-based study of the COVID-19 pandemic, including factors impacting risk of infection and hospitalization. The CCRP includes a subset of immunocompromised (IC) participants with varying vaccination status over time.

**Methods:**

We conducted an observational cohort study of 2,515 IC and 41,941 non-IC CCRP participants who contributed electronic health record data and daily electronic surveys to self-report COVID-19 symptoms, test results, and vaccinations from April 2020 to March 2022. The IC population included those with stem cell transplant, HIV, cancer, autoimmune disease, or solid organ transplant. The latter 3 must have also had an active systemic therapy to meet the IC condition (e.g. chemotherapy, immune modulator, steroid). Logistic regression was used to investigate risk of COVID-19 and hospitalization among IC participants and according to vaccine status within viral variant time periods (pre-delta, delta, omicron).

**Results:**

IC conditions included cancer (51%), autoimmune (41%), solid organ/stem cell transplant (9%), and HIV (7%). The IC group was older and had more comorbidities. 95% of vaccine recipients received an mRNA vaccine. More vaccine breakthrough infections occurred in the IC group than non-IC group (36.1% vs 29.5%, p< 0.001). IC participants were less likely to remain COVID-19 free over time if vaccinated but not boosted (Fig 1A). However, after receiving a booster there was no difference in COVID-19 cases between the groups (Fig 1B). IC participants were more likely to be hospitalized with COVID-19 (OR 2.85; 95% CI 1.69–4.76), but vaccination reduced risk for hospitalization (OR 0.26; 95% CI 0.08–0.8). Receipt of a booster dose reduced risk of COVID-19 among IC participants during the delta wave (IRR 0.52; 95% CI 0.28–0.94) but not during omicron. However, during omicron risk of hospitalization in the IC group was reduced by a booster dose (OR 0.13; 95% CI 0.02–0.72).

**Conclusion:**

IC individuals were at increased risk for COVID-19 hospitalizations and breakthrough infections. After receiving a booster, IC participants were conferred the same level of protection from infection as their non-IC counterparts, highlighting the importance of boosters for these individuals.

**Disclosures:**

**All Authors**: No reported disclosures.

**Figure 1.**
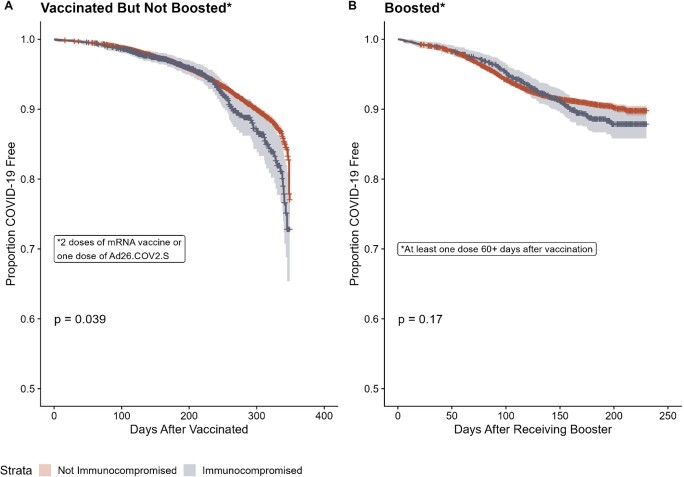
Proportion Remaining Free of COVID-19

